# Air speeds of migrating birds observed by ornithodolite and compared with predictions from flight theory

**DOI:** 10.1098/rsif.2013.0419

**Published:** 2013-09-06

**Authors:** C. J. Pennycuick, Susanne Åkesson, Anders Hedenström

**Affiliations:** 1 School of Biological Sciences, University of Bristol, Bristol BS8 1UG, UK; 2 Department of Biology, Centre for Animal Movement Research, Lund University, Ecology Building, 223 62 Lund, Sweden

**Keywords:** air speed, migration, ornithodolite, wing tips, body drag

## Abstract

We measured the air speeds of 31 bird species, for which we had body mass and wing measurements, migrating along the east coast of Sweden in autumn, using a Vectronix Vector 21 ornithodolite and a Gill WindSonic anemometer. We expected each species’ average air speed to exceed its calculated minimum-power speed (*V*_mp_), and to fall below its maximum-range speed (*V*_mr_), but found some exceptions to both limits. To resolve these discrepancies, we first reduced the assumed induced power factor for all species from 1.2 to 0.9, attributing this to splayed and up-turned primary feathers, and then assigned body drag coefficients for different species down to 0.060 for small waders, and up to 0.12 for the mute swan, in the Reynolds number range 25 000–250 000. These results will be used to amend the default values in existing software that estimates fuel consumption in migration, energy heights on arrival and other aspects of flight performance, using classical aeronautical theory. The body drag coefficients are central to range calculations. Although they cannot be measured on dead bird bodies, they could be checked against wind tunnel measurements on living birds, using existing methods.

## 1. Introduction

We tracked 31 species of birds flying along the east coast of Sweden during the autumn of 2012, in an attempt to determine whether their air speeds were consistent with the predictions of flight mechanics theory. The background to this theory is in the book by Pennycuick [1]. It begins by calculating the rate at which the muscles have to do mechanical work (i.e. the *mechanical power* required) to fly horizontally at a steady speed, relative to the air through which the bird is flying. At slow air speeds, a large amount of power is needed to support the bird's weight against gravity, but this decreases at higher speeds. Another component of power, which is required to overcome the drag of the body, is small at low speeds, but builds up with increasing speed. There are other components, but these two together cause the curve of total mechanical power versus air speed to exhibit a *minimum-power speed* (*V*_mp_), at which the muscles have to do work at a lower rate than at either faster or slower speeds.

### 1.1. Mechanical and chemical power curves

Calculating the curve of mechanical power versus speed is a problem in aerodynamics only, and does not involve physiology. For studies involving fuel consumption, as in long-distance migration, a second power curve is needed for the *chemical power*, i.e. the rate at which fuel energy is consumed in aerobic, horizontal flight. This is derived from the mechanical power curve by first dividing by the efficiency with which the muscles convert fuel energy into work, and then adding some further components of chemical power, especially the basal metabolic rate. Besides *V*_mp_, which is the same for both the mechanical and the chemical power curves, the chemical power curve exhibits a *maximum-range speed* (*V*_mr_), which is higher than *V*_mp_, and is the speed at which the bird covers the greatest distance (relative to the air) per unit fuel energy consumed. In our later analysis, we did not calculate power as such, but only the two characteristic speeds *V*_mp_ and *V*_mr_. The value assumed (0.23) for the efficiency with which the muscles convert fuel energy into work comes from two classical experiments on wind tunnel birds [2,3], and this value affects the estimated chemical power but, perhaps counterintuitively, it has no effect on estimates of *V*_mp_ or *V*_mr_. Basal metabolism does affect *V*_mr_, but it is a minor component of the total chemical power in medium-sized and large birds in cruising flight. For want of a better assumption, we follow tradition by estimating it from regressions based on empirical studies of birds sitting in respirometers, and assume that it continues at the same rate whatever the bird is doing, whether it is active or not, and must be added to the chemical power required for flight.

Power curves for particular birds can be calculated using the program *Flight* 1.24, which is available (free) from http://books.elsevier.com/companions/9780123742995. The program requires morphological information about the bird as input, together with gravity, air density, and some quantities from classical aerodynamics, which are assumed to be species-independent and are assigned default values. By looking for discrepancies between measured cruising speeds and the predictions of the theory, we can reconsider the range of values previously assumed for two of these variables, the induced power factor and the drag coefficient of the body. This in turn increases the confidence with which the *Flight* program can be used for more ambitious projects, such as monitoring the fuel state of migrating birds by analysing GPS data from satellite tracks [4].

### 1.2. Variation of the power curve with body size

**Figure 1.**
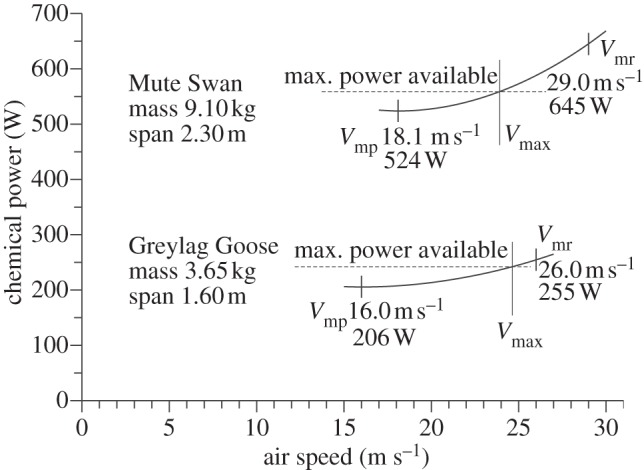
Power curves calculated for sea-level air density from *Flight* 1.24, using mass and wing span from table 1, and values of *k* = 0.90 for the induced power factor and *C*_db_ = 0.10 for the body drag coefficient. The maximum chemical power available depends on the aerobic capacity of the heart and lungs, which is unknown, but is likely to provide a wider margin above minimum power in smaller birds, and very little margin in swans.

Figure 1 shows calculated chemical power curves for two of the larger species in our study, the Mute Swan (*Cygnus olor*) and the Greylag Goose (*Anser anser*) flying at sea level, each marked with *V*_mp_ and *V*_mr_. The mass estimate that we have for the Mute Swan is 2.49 times heavier than that for the Greylag Goose and its wing span is 1.44 times larger than that of the goose. As a result of these two differences, our estimate for *V*_mp_ is 13 per cent faster for the swan than for the goose, and the power required to fly at *V*_mp_ (which is the minimum power required to fly at all) is 155 per cent larger for the swan than for the goose. *V*_mr_ for each species is higher than *V*_mp_ and is defined as the speed where the effective lift-to-drag ratio passes through a maximum. Our estimate of *V*_mr_ is 12 per cent faster in the swan than in the goose and would be the speed at which each species covers the greatest air distance per unit fuel energy consumed, if it had sufficient power to fly at that speed. The maximum speed for level flight is determined by the aerobic capacity of the heart and lungs, which is unknown. However, there is a well-known scaling relationship [1] that results in very large birds, such as swans, having only just enough power to fly at speeds near *V*_mp_, whereas smaller birds have a wider power margin, which allows them to vary their speeds over a wider range.

Being from the same family (Anatidae), the Mute Swan and Greylag Goose are quite close to geometrical similarity, so the differences between the two power curves deviate only a little from those that would result from simply scaling up the goose by linear factor of 1.44. In addition to waterfowl, our 31 study species (table 1) include waders, gulls, terns, raptors, a heron, a cormorant and two passerine species. They cover a mass range of over 400 : 1 and a wing span range of nearly 9 : 1. In general, larger size moves the power curve upwards (higher power) and to the right (higher speeds) as in figure 1, but variations of air density, mass and wing morphology (especially wing span) modify the power curves for different species.

**Table 1.** List of study species, their measurements, and mean equivalent air speeds. Air speed is the mean of run means, and *n* is the number of runs.

species		body mass (kg)	wing span (m)	wing area (m^2^)	air speed (m s^−1^)	s.d. air speed (m s^−1^)	*n*
*Sturnus vulgaris*	Starling	0.0850	0.384	0.0251	15.4	1.71	33
*Motacilla alba*	Pied wagtail	0.0195	0.261	0.0127	13.3	0.810	13
*Falco tinnunculus*	Kestrel	0.229	0.771	0.0791	12.6	2.34	6
*Accipiter gentilis*	Goshawk	0.754	1.05	0.177	16.1	1.57	2
*Haliaeetus albicilla*	White-tailed eagle	4.00	2.19	0.713	14.4	1.04	13
*Ardea cinerea*	Grey heron	1.21	1.60	0.358	12.7	1.71	3
*Cygnus olor*	Mute swan	8.94	2.30	0.683	17.5	1.21	10
*Anser anser*	Greylag goose	3.65	1.60	0.333	19.0	1.93	22
*Anser albifrons*	White-fronted goose	2.45	1.38	0.239	17.8	2.37	10
*Branta leucopsis*	Barnacle goose	1.70	1.34	0.213	17.4	2.08	64
*Branta bernicla*	Brent goose	1.38	1.10	0.143	16.4	1.77	53
*Anas platyrhynchos*	Mallard	1.14	0.890	0.107	19.7	1.55	21
*Anas crecca*	Teal	0.231	0.597	0.0448	17.4	1.60	55
*Anas penelope*	Wigeon	0.770	0.822	0.0829	18.5	2.28	86
*Clangula hyemalis*	Long-tailed duck	0.636	0.690	0.058	19.7	1.70	13
*Somateria mollissima*	Eider	1.91	0.978	0.131	19.0	1.63	25
*Mergus serrator*	Red-breast merganser	0.908	0.860	0.0767	20.0	1.69	34
*Phalacrocorax carbo*	Cormorant	2.56	1.35	0.224	17.4	1.40	52
*Gavia stellata*	Red-throated diver	2.31	1.15	0.128	20.6	1.47	12
*Limosa lapponica*	Bar-tailed godwit	0.200	0.748	0.0568	14.4	1.97	6
*Calidris canutus*	Red knot	0.118	0.516	0.0293	16.1	3.51	4
*Calidris alpina*	Dunlin	0.0477	0.346	0.0147	16.1	1.13	17
*Philomachus pugnax*	Ruff	0.0895	0.472	0.0281	16.9	1.81	8
*Charadrius hiaticula*	Ringed plover	0.0618	0.384	0.0169	16.0	1.07	4
*Pluvialis squatorola*	Grey plover	0.258	0.630	0.0437	16.5	1.76	8
*Haematopus ostralegus*	Oystercatcher	0.403	0.852	0.0873	15.9	0.564	3
*Sterna hirundo*	Common tern	0.131	0.781	0.0507	11.0	1.83	21
*Larus ridibundus*	Black-headed gull	0.282	0.962	0.0982	11.4	1.47	36
*Larus canus*	Common gull	0.404	1.10	0.138	12.9	1.47	30
*Larus argentatus*	Herring gull	0.705	1.35	0.200	13.4	1.37	47
*Larus fuscus*	Lesser black-backed gull	0.818	1.34	0.190	14.4	1.34	7

### 1.3. Conditions for valid comparisons

**Figure 2.**
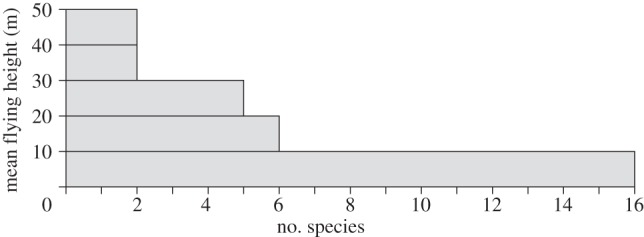
Mean flying heights above the water surface for the 31 species in our sample.

Our assessment of measured speeds against the predictions of theory is only as reliable as the mass and wing measurements that we used to calculate the power curves for each species. We did not use data of doubtful reliability from the literature, and dropped several species from the analysis despite having enough tracks, because we did not have mass and wing measurements from a trusted source. Comparison of observed speeds with calculated characteristic speeds also depends on the birds' behaviour being close to steady flapping flight at a constant speed and height, as those are the conditions for which the power curve is calculated. To make the field data as homogeneous as possible, we measured the speeds of birds migrating along the shore, including only birds that were judged by the observer (A.H.) to be flapping steadily along, with minor changes of direction and height. Figure 2 shows that the average flying height was less than 50 m above the water surface in all 31 species in our sample, and less than 10 m in 16 of them. As most tracks were several hundred metres long, these low flying heights constrained the flight paths to be nearly horizontal, as assumed by the theory.

## 2. Material and methods

### 2.1. Triangle of velocities

We measured the bird's air speed, for comparison with the predictions of theory, in two stages. First, we measured the *ground speed vector*, consisting of the bird's speed relative to the observer's position on the shore and its track direction, i.e. the direction in which the bird was moving over the ground, measured clockwise from true north. We also measured the *wind vector* consisting of the wind speed and the direction from which the wind was blowing, and then obtained the *air speed vector* (air speed and heading direction) by vector subtraction of the wind vector from the ground speed vector [1]. The bird's heading is the direction in which it is steering, and the *drift angle* is the difference between the heading and the track.

### 2.2. Ground speed measurement

Our tracking instrument was a Vectronix Vector 21 Aero, which is a tripod-mounted pair of 7 × 42 binoculars with three built-in sensors, a laser rangefinder, a magnetic compass and an angular elevation sensor. With the addition of a computer (Fujitsu Lifebook) to record the data and provide a timing source, the Vector can be used as an ornithodolite, as defined by Pennycuick [5,6]. When tracking a bird, simultaneous readings from all three sensors were sent via the Vector's serial output to the computer, and combined with the time from the beginning of the run (to 0.1 s) from the computer's real-time clock. We call this record an ‘Observation’ of the bird's timed, three-dimensional position in space, with the observer at the origin. A series of two or more observations on the same bird is called a ‘Run’. Ground speed and vertical speed were found, respectively, from the horizontal and vertical distance between each observation and the one before. We wrote custom software in Visual Basic .NET, developed from a previous Vector ornithodolite project in which the earlier Vector 1500 was used [7]. The Vector 21's rangefinder proved to be much better than that of the Vector 1500, and routinely allowed us to start tracking ducks and geese when they were over 2 km away. It had no difficulty in tracking black birds such as cormorants, which had been a problem with the Vector 1500, and its recovery time after taking an observation was about 2 s, notably quicker than the Vector 1500.

**Figure 3.**
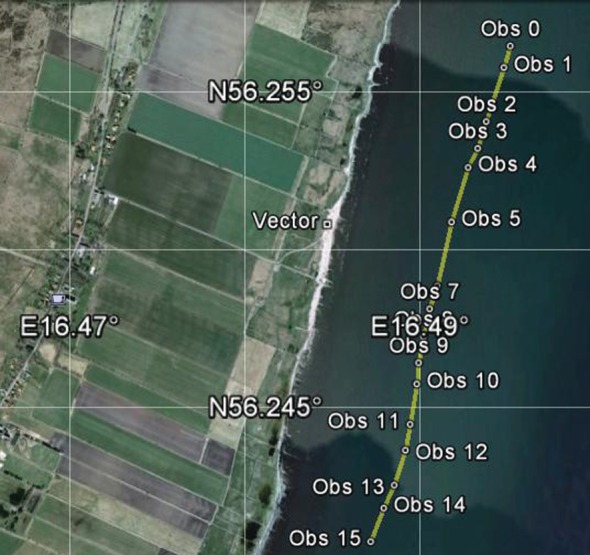
Track of a Brent Goose, one of a flock of 36, shown in Google Earth from the KML file. The observer's position on the shore is marked ‘Vector’, and the anemometer was nearby. This goose was tracked for 2 min 16.8 s, for a ground distance of 1760 m. There were 16 observations (0–15) giving 15 air speed estimates, which were averaged to get the Run Mean and s.d. for the air speed (14.5 ± 0.953 m s^−1^). The wind, from a single anemometer reading immediately after the run, was 2.3 m s^−1^ from 246° (True), measured at 7.2 m above the water surface. The corrected wind (used to calculate the air speed) varied between 1.4 and 2.5 m s^−1^ as the goose's flying height varied between 1.1 and 10.1 m. This track is typical of 53 that were obtained for this species. (Online version in colour.)

Errors can occur with the Vector if the rangefinder pulse misses the bird and is reflected instead by a foreground or background object. We were usually aware of such errors when they occurred, but we also generated a KML file for each run, which allowed us to display the bird's track later in Google Earth, superimposed on a map of the coastline (figure 3). Bad observations were easily detected as points on the track that were displaced along the Vector's line of sight, and could be deleted from the file. We examined the KML file for every run in our study, and deleted the few bad observations that we found.

### 2.3. Wind measurement

**Figure 4.**
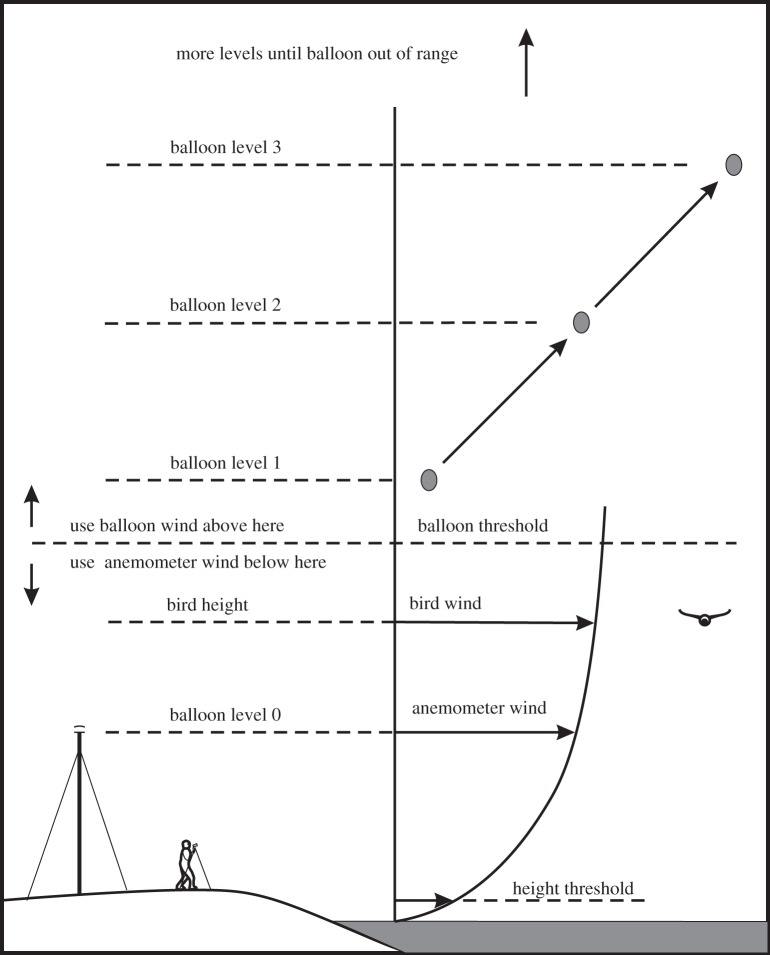
Observing set-up and wind sources.

Wind measurement was a crucial part of our observations, and was facilitated by our choice of a coastal observing site at Näsby on the east side of Öland, Sweden (56°15.1′ N, 16° 29.1′ E) with low-lying land to the west, the Baltic Sea to the east and no nearby buildings or trees to cause turbulence. For low flying birds, we measured the wind with a Gill Windsonic anemometer mounted on a 5-m mast in an unobstructed location near the ornithodolite, and transmitted the reading to the computer at 1-s intervals, via a pair of wireless modems (Haccom UM-96). As there was no discernible tidal variation of the water level against rocks along the shore, and wave amplitude was minimal even in wind speeds up to 12 m s^−1^, we were able to get a meaningful measurement of the anemometer height above the water surface (*h*_an_) by measuring the Vector's height above the surface, and also the anemometer's height above the Vector. We took these two measurements as part of the set up procedure at the beginning of each observing session. The current anemometer reading was automatically recorded as part of the data for each run, and later corrected for surface friction, according to the bird's height above the surface. This was done in two steps according to the procedure of Ruggles [8], which was developed for light wind and sea conditions, similar to those that prevailed during our study. The friction wind (*V*_fr_) was first calculated as2.1
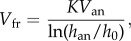
where *V*_an_ is the anemometer reading at a height *h*_an_ above the surface, *K* is von Karman's constant (0.42) and *h*_0_ is the roughness height, taken to be 5 cm. Then, the wind speed *V*_w_ at the bird's measured height (*h*) was found as2.2
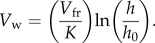
This results in a wind curve which starts at zero at a height *h*_0_ above the surface, passes through *V*_an_ at the anemometer height, and continues to increase above that, ever more gradually (figure 4). The *height threshold* was set for each run to half the bird's wing span. If the height reading from the Vector was below the height threshold, we calculated the wind as though the bird were at the threshold, not below.

A second boundary, the *balloon threshold*, was set at 15 m above the surface. This is an arbitrarily chosen level, above which we used a wind estimate derived from balloon ascents instead of extrapolating from the anemometer wind. Before and after each observing session, and at intervals of approximately 1 h during the session, we released a helium-filled balloon and tracked it with the Vector, recording a run as though it were a bird. Each balloon ascent was later analysed to produce a wind profile, consisting of a stack of estimates of wind speed and direction, one for each observation of the balloon, except that the bottom layer (level 0) came from the anemometer. During later analysis, the wind speed and direction were interpolated between the balloon ascents before and after each observation, to obtain an estimate of the wind speed and direction at the bird's height and the time of the observation.

The balloon ascents allowed us to get wind estimates up to the maximum height at which we could track a balloon, which itself depended on the wind strength. We twice tracked balloons to over 500 m in light winds (5 m s^−1^), but more often had to be content with heights of 100–200 m, before the wind carried the balloon beyond the Vector's range. The interpolation technique resulted in neglecting short-period variations in the wind, whereas the anemometer provided a wind estimate immediately after each run, but only for birds that were flying below the balloon threshold. The use of anemometer and balloon measurements is summarized in figure 4.

### 2.4. Air density

We recorded the ambient air temperature and pressure at the observer's position at the beginning of each session, and updated these values as necessary between runs. The mean air density for all sessions in the study was 1.23 kg m^−3^ (s.d. 0.0145 kg m^−3^). This is indistinguishable from the sea-level density in the International Standard Atmosphere (1.225 kg m^−3^). An estimate of the air density at the bird's measured flying height was computed [1] for each observation and recorded as part of the data.

### 2.5. Bird identification and multi-species flocks

No useful hypotheses can be tested without identifying each bird that is tracked, but this was not usually a problem as the observer had an excellent view of the bird through the Vector. We recorded the species and some other details as part of the data for each run. We also used a telescope to identify approaching birds, before they were within range of the Vector's rangefinder, and were able to gather data more quickly if a skilled spotter was available to do this, while the observer was entering details of the last bird tracked.

The waterfowl and waders often flew in compact flocks, often with more than one species in the flock. In this case, we identified the species as the one with most individuals in the flock, assuming, in effect, that minority species would adjust their speed to that set by the majority. We recorded the number of birds in the flock (all species) as part of the data for the run, so that we could test whether it had any effect on the speed.

### 2.6. Wing measurements

Calculating characteristic speeds calls for body mass and wing measurements to be made according to standard definitions. *Wing span* is the distance from one wing tip to the other, with the wings extended out to the sides as far as they will go, with the elbow and wrist joints fully extended. *Wing area* is the projected area of both wings, similarly extended, including the area of the body between the wing roots. Practical measurement procedures are given by Pennycuick [1]. All of the measurements that we used, and many others, can be found in the Wings Database that comes with the program *Flight* 1.24.

## 3. Results

### 3.1. Observed mean air speeds

**Figure 5.**
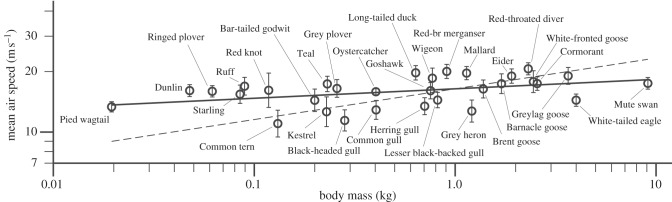
Double-logarithmic plot of equivalent air speed (mean of run means) versus body mass. The error bars are the s.d. of the run means. The slope is 0.047, meaning that the speed varies, on average, with the 0.047 power of the body mass. The dotted line shows the expected slope (0.153) for *V*_mp_ versus body mass, taking account of known wing allometry [1].

We recorded 951 runs on 83 species, observing from the same site (above) on 19 days during a two-month period from 4 September to 2 November 2012. From these, we selected a subset of 31 species (table 1) for which we had mass and wing measurements, and also enough runs to calculate a mean and standard deviation for the mean air speeds from the individual runs. Figure 5 shows a double-logarithmic plot of the observed mean speeds versus body mass. These are equivalent air speeds, which have been reduced to sea level, by multiplying the measured true air speed by the square root of the ratio of the ambient density to the sea-level value in the International Standard Atmosphere. As noted in §2, a run of *N* observations yields *N* – 1 estimates of air speed. As these were selected runs in which the bird was flying steadily along, a mean speed could be calculated, which was reasonably representative of the run. Each point in figure 5 is the mean of all the run means for one species, in which the bird was scored as flying straight, and either flapping, intermittently flapping and gliding, or bounding. The error bars are the standard deviations of the run means. The linear regression line shows a small positive slope of 0.047, meaning that the observed speed varied with the 0.047 power of the body mass, although the correlation coefficient (0.414 for 31 points) is barely significant. The slope is, however, significantly less than that of the dotted line (0.153; *t*-test *p* < 0.001), which was obtained by calculating *V*_mp_ for all the species in the Wings Database that comes with the *Flight* program, and plotting it against body mass, so taking account of wing span allometry [1]. This means that the birds that we tracked did not fly at a constant multiple of *V*_mp_. The ratio of air speed to *V*_mp_ in this sample was larger in small species than in large ones.

### 3.2. Ratio of air speed to *V*_mp_

**Figure 6.**
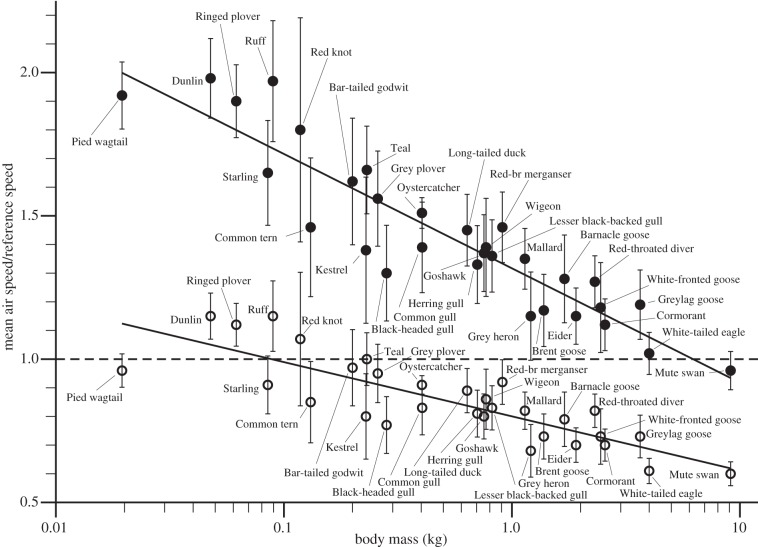
Log-linear plot of the ratio of the mean air speed (*V*_a_) to a ‘Reference speed’, which is *V*_mp_ for the upper line (filled circles), and *V*_mr_ for the lower line (open circles). The horizontal dotted line represents *V*_a_/*V*_mp_ = 1 for the solid circles, and *V*_a_/*V*_mr_ = 1 for the open circles. The filled circles are not expected to fall below the dotted line, nor open circles above it.

Figure 6 shows the ratios of the mean air speed observed for each species to our estimates of the two characteristic speeds predicted by theory, *V*_mp_ and *V*_mr_, as illustrated in figure 1. These ratios were calculated for each individual observation, and once again the mean of the run means has been plotted versus body mass. A log-linear plot has been used, although the regression lines are shown only as a qualitative indication of the trend, as we do not have a hypothesis that would predict a logarithmic relationship. The downward trend is due to a well-known scale effect, whereby the mechanical power available from the muscles scales differently from the minimum power required to fly horizontally [1]. This implies that while small and medium-sized species have sufficient power to fly at a range of speeds from *V*_mp_ up to some maximum that is well above *V*_mp_, larger species are confined to speeds only just above *V*_mp_, and there is an upper limit to the mass at which a bird has enough power to fly horizontally at all (at *V*_mp_). Still heavier birds are possible, but cannot maintain height in flapping flight, and this may be true of condors and the largest albatross species.

A bird such as the Mute Swan, which is near the upper limit of mass for level flight, has a small amount of power in reserve when flying at *V*_mp_, and it might be thought from the U-shape of the power curve that such a bird would be able to use this reserve to fly either slightly faster than *V*_mp_ or slightly slower. Flying slower than *V*_mp_ is possible, and some birds (flycatchers, kingfishers and hummingbirds) are even specialized to do this, but it is difficult because speeds below *V*_mp_ are unstable. No migrating bird cruises at a speed that is even marginally below *V*_mp_, because if it tries to do that, it has to exert more power than would be needed at a slightly higher speed, and therefore tends to speed up. Any small disturbance causes the bird to accelerate through *V*_mp_, until the speed automatically stabilizes on the rising part of the power curve, above *V*_mp_, where the power required is the same as before [1]. The horizontal dotted line for *V*_a_/*V*_mp_ = 1 is therefore an absolute lower boundary for the distribution of the points. The solid circles in figure 6 are species averages, and we did not expect any of them to fall below this line. However, when we calculated *V*_mp_ with the existing default values in the *Flight* program, the observed values of air speed were below the respective estimates of *V*_mp_ for the two largest species, the White-tailed Eagle and the Mute Swan, which suggested to us that we needed to re-examine the default values used in the program (below).

### 3.3. Ratio of air speed to *V*_mr_

Since *V*_mr_ is the speed at which a migrating bird covers the greatest air distance per unit fuel energy consumed, we also did not expect any of the open circles in figure 6 to fall *above* the dotted line, meaning that the average air speed was faster than *V*_mr_. Hedenström & Alerstam [9] have argued that cruising speeds above *V*_mr_ may be optimal in some circumstances, even though this requires increased aerobic capacity, and this may be the explanation for the four small waders (Dunlin, Ringed Plover, Ruff and Red Knot) that show average air speeds above *V*_mr_ in figure 6 (open circles above the dotted line). Alternatively, we may have underestimated *V*_mr_, possibly in all species, and we can check what the implications of that would be, in terms of the variable values that we used for calculating *V*_mr_.

## 4. Discussion

### 4.1. Estimating *V*_mp_

The formula that we used to estimate *V*_mp_ is4.1
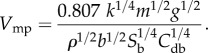
The derivation is given by Pennycuick [1]. Wrong values of any of the variables on the right-hand side of equation (4.1) will result in either an under or an overestimate of *V*_mp_, with corresponding errors in the ratios plotted in figure 6. The values of five of these seven variables were known or measured, and we first briefly review these as possible sources of error. The remaining two variables are the induced power factor (*k*) and the drag coefficient of the body (*C*_db_). These are difficult to measure and are assigned default values in the *Flight* program, which may need revision in the light of our results.

### 4.2. Known or measured variables

*Gravity* (*g*) is assigned the value 9.81 m s^−2^, which is within 0.5 per cent of the actual value, anywhere that birds go [1].

*Body mass* (*m*) was not known for individual birds, but samples of measurements were available for each species that featured in the analysis, and the means of these samples were used to calculate *V*_mp_. This would bias the estimate of *V*_mp_ downwards for a bird that was lighter than assumed, and might bias a sample downwards if the birds were low on fat, at the end of a long non-stop stage. However, this was not the case for any of the migrants passing our study site on the east coast of southern Sweden.

*Wing span* (*b*) also came from the mean of a sample of measurements of each species, but does not vary in individuals in the way that body mass does. Samples of a species typically show a standard deviation of 3 per cent of the mean. We used our own data for wing spans.

*Air density* (*ρ*) was measured at the observer's position and updated during each observing session. An estimate of the air density at the bird's measured flying height was recorded as part of the data for each observation, according to the height correction in [1].

*Body frontal area* (*S*_b_) was estimated from the body mass using the following formula:4.2
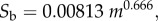
which comes from measurements of frozen bird bodies used in wind tunnel experiments by Pennycuick *et al*. [10]. The effect of the body frontal area on *V*_mp_ cannot be distinguished experimentally from that of the drag coefficient, because both affect the result in the same way in equation (4.1). The body frontal area is difficult to measure repeatably, so we assume in effect that the body shape is the same in all birds, and attribute variations in body drag to the drag coefficient only.

### 4.3. The induced power factor for wings with upturned tips

The induced power factor (*k* in equation (4.1)) is the factor by which the induced power exceeds the value for an ideal actuator disc, in which a constant downwash velocity is produced over the whole of the disc area, stopping abruptly at the edge of the disc [1]. Revising the value of *k* used in equation (4.1) downwards would lower the low-speed end of the power curve, so reducing the estimate of *V*_mp_. The ideal induced power (*k* = 1) is actually the same whether the wings sweep out the entire disc as in a helicopter rotor, or part of it as in flapping wings, or none at all as in a fixed wing [11]. The fixed-wing case is the familiar one of a *planar* wing with an elliptical lift distribution. Practical wings can approximate at best to the ideal lift distribution, and consequently it is widely believed that *k* = 1 represents the lowest induced power attainable with a fixed wing or helicopter rotor, and that real wings have values of *k* that slightly exceed 1. However, the classical Prandtl lifting-line theory (presented in aeronautical textbooks such as Anderson [12] and von Mises [13]) does not exclude the possibility that *non-planar* wings, in which the wing tip is bent upwards, can make the wing behave as though its span were longer than it actually is, thus reducing the induced power. Reductions in the induced drag of airliner wings equivalent to *k* = 0.88 have been measured at cruising speeds (lift coefficient 0.6) in airliners with modified wing tips that bend upwards [14], and this effect would be stronger at lower speeds and higher lift coefficients.

Berens [15] has comprehensively considered not only the induced drag, but also the pressure drag and skin friction of a wide variety of non-planar wing shapes, including several that were inspired by the wings of large birds such as storks and vultures, in which the emarginated primary feathers separate and bend upwards in gliding flight, to form a cascade of small, non-planar aerofoils around the wing tip. He estimated that values around *k* = 0.8 would be typical for this kind of wing. The effect works by displacing the cores of the wing tip vortices outwards, so that the wing imparts downwash to a wider swath of air than it would with planar tips. Dr Heinrich Eder (2013, personal communication) has observed such an outward displacement behind the wing of a White Stork (*Ciconia ciconia*), when mounted in the Seewiesen low-turbulence wind tunnel, in an air flow that bends the primary feathers into approximately the configuration seen in flight, and he estimates that this would lead to a value of *k* = 0.9 or even less, depending on the lift coefficient.

**Figure 7.**
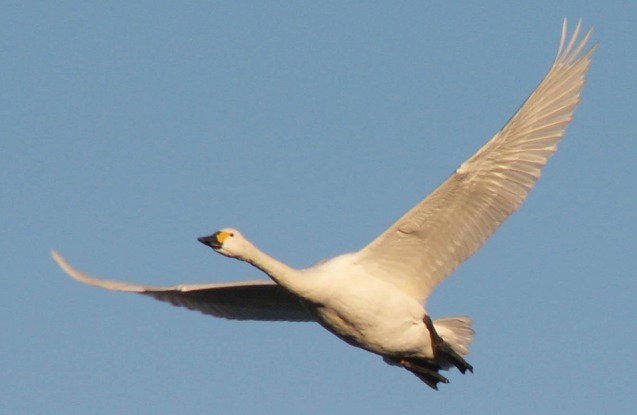
Bewick's Swan (*Cygnus columbianus*), showing separation and upward bending of the primaries at the wing tip, when the wing is highly loaded in the downstroke of flapping flight, and the lift coefficient is high. The Mute Swans in our study also held their feet in the same position when flying, below the tail. (Online version in colour.)

We are concerned mainly with the wings of swans, ducks and waders, which are more strongly tapered than those of storks with narrower tips. However, their primary feathers also separate at the tips into a cascade of small, up-turned winglets during the downstroke of flapping flight, when the lift coefficient is high, and the induced power is required (figure 7). All the species in our sample have wing tips that splay in this way in flapping flight, and this appears to be a general feature of all flying birds, including pointed-winged species such as albatrosses, falcons and swifts. By analogy with a fixed wing with winglets, the effect would be that the wing sweeps out an effective disc area that is larger than it would be with simple planar tips, so reducing the induced power. It follows that the original default value for the induced power factor in the *Flight* program (*k* = 1.2) is not realistic, and should be revised downwards. Figure 6 was originally calculated with the *Flight* program's current default values, *k* = 1.2 and *C*_db_ = 0.10, and with these values (not shown) both the Mute Swan and the White-tailed Eagle showed average speeds less than *V*_mp_. This suggests that the estimate of *V*_mp_ was too high and needs to be reduced either by reducing *k* or increasing *C*_db_ (equation (4.1)). To calculate the points in figure 6 (as shown), we reduced *k* to 0.90, and kept *C*_db_ unchanged at 0.10. Now the solid circle for the White-tailed Eagle is above the line, but that for the Mute Swan is still below. Although a still lower value of *k* is not ruled out, we turned at this point to the body drag coefficient, which also affects the estimate of *V*_mp_.

### 4.4. Body drag coefficient

Revising the value used for the body drag coefficient (*C*_db_) in (equation 4.1) upwards would raise the high-speed end of the power curve, so decreasing the estimate of *V*_mp_. It might appear that *C*_db_ can easily be measured by mounting a frozen or stuffed bird body on a drag balance in a wind tunnel, and many authors (ourselves included) have done this. The results are always anomalously high, in the region of 0.2–0.4, which is associated with bluff bodies rather than streamlined bodies. It is now known that this is an artefact, caused by massive separation of the boundary layer from dead bird bodies, which does not occur in living birds. Measurements on living birds flying in the Lund wind tunnel, in which *V*_mp_ was measured, and *C*_db_ was deduced by inverting equation (4.1) [16], gave an estimate of *C*_db_ = 0.08 for both a Teal and a Thrush-Nightingale, and later measurements by the same method on Rose-coloured Starlings that were flying in the Seewiesen wind tunnel [17] gave a mean *C*_db_ of 0.12. If *k* = 0.9, the Mute Swan's body drag coefficient has to be increased from 0.10 to 0.12, to bring the estimate of *V*_mp_ below its observed mean air speed.

We were not able to determine the sex of Mute Swans that we tracked and were concerned that the unknown sex ratio might have biased our estimate of *V*_mp_ in this strongly dimorphic species. However, we had measurements (in the Wings Database included with the *Flight* program) of mass and wing span in a sample of eight males and eight females, in which the sex was determined by cloacal examination during routine winter swan catches at the Wildife and Wetlands Trust at Caerlaverock. Females are lighter than males, which reduces the estimate of *V*_mp_, but they also have shorter wing spans, which increases *V*_mp_. Table 2 shows that the observed mean equivalent air speed (17.5 m s^−1^) is still below the estimated *V*_mp_, even if the observed swans are assumed to have been all females.

**Table 2.** Means and standard deviations of measurements of Mute Swans of known sex from winter swan catches at the Wildfowl and Wetlands Trust, Caerlaverock.

sub-sample	*n*	mean mass (kg)	mean wing span (m)	mean air speed (m s^−1^)	ratio air speed : *V*_mp_
sexes combined	16	8.94 ± 1.26	2.30 ± 0.111	17.5 ± 1.21	0.969 ± 0.0672
adult males	8	10.05 ± 0.69	2.40 ± 0.044	17.5 ± 1.21	0.953 ± 0.0661
adult females	8	7.83 ± 0.47	2.21 ± 0.046	17.5 ± 1.21	0.992 ± 0.0688

### 4.5. Birds flying faster than *V*_mr_

Because of its physiological component, the estimate of *V*_mr_ has to be computed numerically from the power curve, rather than calculated from a formula like *V*_mp_, but it too decreases if the value of *k* is decreased, and increases if *C*_db_ is decreased. Being a higher speed, *V*_mr_ is less strongly affected by changes in *k* than *V*_mp_, but more strongly affected by changes in *C*_db_. The combination of *k* = 0.9 and *C*_db_ = 0.10 leaves the four small wader species, noted earlier, apparently cruising at speeds above *V*_mr_ (open circles in figure 6). This second anomaly would be resolved with the same value of *k* (0.9), if *C*_db_ for these species were no more than 0.078 for the Red Knot, 0.066 for the Ringed Plover and 0.060 for the Dunlin and the Ruff. If the body drag coefficients of these species were shown to be higher, then we would have to invoke the optimality argument put forward by Hedenström & Alerstam [9], although it may be noted that there are other uncertainties in the calculation of chemical power that could affect estimates of *V*_mr_ [1].

### 4.6. Reynolds number and body drag

The Reynolds number in cruising flight, based on body diameter, would be in the range 25 000–40 000 for the four wader species and around 250 000 for the Mute Swan, for which we propose body drag coefficients of 0.060–0.078 and 0.12, respectively. If this represents a trend, it is in the opposite direction from that expected. Drag coefficients for bodies of similar shape normally increase at lower Reynolds numbers in this range, because skin friction contributes a larger fraction of the total drag at the lower end of the range, and also because there is an increased tendency for the boundary layer to separate from the surface. On the other hand, the morphology of feet and tails is expected to lead to species-specific variations in the body drag coefficient [16]. Waders have bodies that taper to a point at the rear end, small tails that can be completely furled in cruising flight, and thin legs that trail below and behind the tail, where they would create minimal drag. Mute Swans may well have higher body drag coefficients than waders, despite their larger size and the higher Reynolds numbers at which they fly, because of their long necks and large feet, which are commonly trailed below the tail in flight (figure 7). If that is so, we would expect to see the same speed anomaly in migrating Whooper Swans (*Cygnus cygnus*), and we hope to check this in the future. It would be especially interesting and relevant to measure the body drag coefficients of any or all of the four wader species, which could be done by the wingbeat-frequency method in the Lund wind tunnel. The *Flight* program defaults can of course be over-ridden by users. We propose leaving the default *C*_db_ = 0.10 where it is, and recommending users of the program to reduce it for birds with especially well-streamlined bodies, such as waders.

## 5. Conclusions

### 5.1. Anomalies and their resolution

The answer to our original question is that our speed measurements did indeed show a pattern that is easily understood in terms of the theory of the power curve, but there were some anomalies, which can be resolved by reducing the induced power factor to a value less than 1, and assigning values ranging from 0.06 to 0.12 to the body drag coefficient for small waders and swans, respectively. By adjusting the default value of the induced power factor in the *Flight* program to *k* = 0.9, and leaving the default body drag coefficient at *C*_db_ = 0.10, we recognize that slotted wing tips with separated, upturned primary feathers effectively increase the wing span, and that species whose body shapes resemble classical streamlined bodies are likely to have lower body drag coefficients than those with prominent heads, big feet or long tails. It is best to adjust the value of *C*_db_ for different species, especially if reliable measurements are available. Drag measurements on dead bird bodies are not reliable, and very few *C*_db_ measurements on living birds have been published, but it is possible to make these measurements with well-trained birds in a high-quality wind tunnel, such as those at Lund and Seewiesen.

### 5.2. Wider implications of the results

The proposed revision of default values in the *Flight* program represents a minor adjustment in assumptions, in the light of new data. By using the program to interpret the results, rather than applying statistical analysis to our speed measurements, we add to the body of data on which the program bases its predictions, which cover a range of topics that might not appear to be closely related to speed measurements. For example, the program was used by Pennycuick *et al*. [4] to provide running estimates of fuel consumption and reserves in migrating geese, using GPS data from satellite tracking. Reducing the induced power factor would slightly reduce the estimates of fuel consumed in flight, and increase the energy heights at which the geese arrived at their destinations, but would not affect the conclusions about their migration strategy. *V*_mp_ and *V*_mr_ were tracked in individual birds on these flights, as they used up fuel and decreased their mass, and varied their height. These geese were only seen flying at speeds approaching *V*_mr_ after they had reduced their weight by consuming a substantial amount of fuel, and they showed other indications that they had little aerobic capacity to spare, especially when crossing the Greenland ice cap. However, it is possible that waders, especially the smaller species, might have enough aerobic capacity to fly faster than *V*_mr_, if they have any reason to do that. Little is known about level-flight performance at speeds around *V*_mr_ or above in any species, and this too could be addressed in wind tunnel studies on the small waders in our study. Our Vector ornithodolite system is, of course, adaptable to field studies of other types of flight besides level cruising, for example, soaring in thermals.
